# Work–Life Balance of the Employed Population During the Emergency Situation of COVID-19 in Latvia

**DOI:** 10.3389/fpsyg.2021.682459

**Published:** 2021-08-06

**Authors:** Jelena Lonska, Iveta Mietule, Lienite Litavniece, Iluta Arbidane, Ivars Vanadzins, Linda Matisane, Linda Paegle

**Affiliations:** ^1^Business and Society Process Research Center, Rezekne Academy of Technologies, Rezekne, Latvia; ^2^Institute of Occupational Safety and Environmental Health, Riga Stradins University, Riga, Latvia

**Keywords:** work–life balance, COVID-19, teleworking, remote working, homework

## Abstract

All the employees face the challenge of finding the right work–life balance. The ability of employees to deal with the successful combining of work, family responsibilities, and personal life is crucial for both employers and family members of employees. During the COVID-19 emergency situation, many people around the world were forced to work remotely. Initially, there were observed some certain expectations about the possibility of working from home as a positive factor that will promote work–life balance. However, over time, negative tendencies were also revealed, as employees were only one call or message away from the employer, and uncertainty and leisure time with family often created more stress. As many organizations and individuals were not ready for this sudden change, many mistakes were made, which further raised the issue of work–life balance. The aim of the research was to evaluate the flexibility of reconciling work and private life of Latvian employees in various socio-demographic groups during the COVID-19 emergency situation in spring 2020, to investigate how family life influenced employees’ ability to perform work duties, to find out if employees had any additional housework responsibilities and how their workload changed concerning housework amount during the COVID-19 emergency situation. The research is based on the data obtained in the survey of the Latvian employed population, which was conducted within the framework of the Latvian National Research Programme Project “CoLife” in the second half of 2020. As a result, the hypothesis of the research that all groups of employees experienced work–life balance difficulties during the COVID-19 emergency situation has been partially confirmed, i.e., women in the 18–44 age group and respondents with minor children in the household more likely faced difficulties of work–life balance. The scientific research methods that were used in the research are the monographic method, content analysis, survey, data processing with SPSS to determine the mutual independence of the data from the questionnaires.

## Introduction

All the employees face the challenge of finding the right work–life balance. The ability of employees to deal with successful combining of work, family responsibilities and personal life is crucial for both employers and family members of employees. Work–life balance not only means an even distribution of time between work and private life, but rather flexibility in being able to work in the professional field, while maintaining the time and energy to spend on personal life.

According to scientific and practical research, one of the tools for work–life balance is remote working, but it must be borne in mind that the COVID-19 emergency situation for employees who have minor children changed the everyday life of private life, responsibilities, and timing. Considering that the emergency situation has encouraged the use of remote working, which has the tendency to increase, it is essential to facilitate/ensure work–life balance for employees, regardless of employment type and form of working hours.

The aim of the research is to evaluate the possibilities of flexibility to reconcile work and private life of Latvian employees in various socio-demographic groups during the COVID-19 emergency situation in spring 2020; to study how family life affected employees’ ability to perform work duties, to find out whether employees had additional housework responsibilities and how their workload changed performing household responsibilities during the COVID-19 emergency situation. The answers were evaluated by gender and age of the respondents, as well as depending on the presence of minor children in the household.

Within the framework of the research, a hypothesis was put forward—during the COVID-19 emergency, all groups of employees experienced difficulties in balancing work and private life.

The significance of the research becomes particularly important in the COVID-19 emergency situation, which has exactly affected the remotely working population, significantly affecting their work–life balance. When working remotely, employees tend to disregard work schedule, because it is difficult to distinguish between working hours and free time, which can lead to a deterioration of employees’ psycho-emotional state and increased tension. In such a situation, it is important for the employers to support the employees—both in the organization of remote working (for example, providing the opportunity to freely plan one’s working hours, introduce changes in work schedules that allow combining work and housework responsibilities) and in providing psycho-emotional support, what in general enhances the work–life balance of employees. As a result, employers obtain increased work efficiency and productivity, improved employee’s health, higher motivation, and strengthened loyalty of the employee, etc.

The novelty of the research is related to the fact that for the first time in Latvia there was conducted a survey of employed population from different socio-demographic groups with the aim to evaluate the possibilities to reconcile their work and private life in order to reduce the spread of COVID-19 during the period of imposed restrictions.

The scientific research methods that were used in the research are monographic method, content analysis, survey, data processing with SPSS to determine the mutual independence of the data from the questionnaires.

## Literature Review

In the EU countries, including Latvia, the balance between employees’ work and private life is becoming more and more important. This issue became particularly important during the spread of the COVID-19 consequences. Consequences of continuous remote working and access to the employer, stress caused by long-term use of technologies, burnout syndrome, the need to take care of children, and sick family members while performing work responsibilities, unavailability of opportunities to look for children—all the factors have a significant impact on work–life balance and quality of life.

The separation of work and private life is a challenge that most of the people active in the labor market face. In many cases, this can lead to burnout—a state of physical and mental exhaustion when a person’s ability to work is drained. It was found that the negative impact of work on work–life balance is usually concentrated in the early stages of parenting, when employees in the household have pre-school children (Eurofound, 2017).

There are different approaches to the use of the concept “work-private life balance,” i.e., equilibrium of work and family life, equilibrium of work and private life, etc. Earlier research often deal with examination of the equilibrium between family and professional life in relation to the concept of work–life balance (Ramakrishnan, 2020a,b). The concepts of “work–life balance” or “work–personal life reconciliation” are widely used to raise awareness of which areas of life need to be combined and reconciled, thereby forming division of work and non-working life, emphasizing that reconciliation is required not only for work and family life, but also religious activities, involvement in community life, education, and other activities (Korpa, 2012).

Work–life balance is formed when a person has the same level of priorities in relation to the requirements of his/her career and the requirements of personal life. The most common reasons for imbalance between the personal life and work life are increased responsibility for work commitments; working longer hours; increased responsibility for housework as well as for employees with children. In turn, a positive work–life balance reduces employee stress, reduces the risk of burnout and creates greater wellbeing. This positively affects not only an employee him/herself but also the employer (Sanfilippo, 2020).

Work–life balance can be viewed much more broadly, considering its positive impact on social and economic sustainability. Improving work–life balance is linked to higher-level goals, including: increasing employment in the labor market, ensuring equal opportunities for the sexes, tackling demographic challenges. Our needs and the needs of our children or dependents change with age. Our necessity for work–life balance is also changing (Parent-Thirion, 2016).

Already at the beginning of this century, remote working was evaluated as an important tool for promoting work–life balance. Remote working enables a variety of family responsibilities and can be particularly useful for employees with children, as it allows them to breastfeed, take care of a sick child or look after young children who may be on school holidays. Regular remote working offers additional advantages, as it reduces work-related expenses (such as travel costs) and saves time spent on the way to work (Hein, 2005).

During the COVID-19 emergency situation, many people in the world were forced to work from home. Initially, there were observed some certain expectations about the possibility of working from home as a positive factor that will promote work–life balance. However, also negative trends appeared, as employees were only one call or message away from the employer, and it was therefore expected that the employee would work outside working hours and would also be available outside working hours. Uncertainty and spending time with family often caused more stress. As many organizations and individuals were not ready for this sudden change, many mistakes were made, which further raised the issue of work–life balance. At the same time, the COVID-19 emergency situation has provided valuable lessons. The public is offered the opportunity to think about what cooperation means in reality and how it can improve collaboration between companies and employees. Employers are facing new challenges; and it is essential to ensure both the economic growth of companies in the future and to create praxis supporting the work–life balance of employees. Work–life balance, especially for an indefinite time, such as caused by COVID-19, is essential for employee growth, personal happiness and company retention. When employees receive support to find a positive work–life balance, they are usually more motivated to do the job qualitatively (Ramakrishnan, 2020a,b).

Some studies conducted all over the world show that most people have not improved their work–life balance during the emergency situation, even though they were able to spend more time with their families and did have to spend time to get to workplace. For most people, the period of COVID-19 emergency situation seemed more stressful as they spent more time in webinars and meetings. They also lacked “chatting” with colleagues. The division between family time and working time overlapped so much that they found it difficult to cope with. Moreover, the uncertainty about work and the future compounded the problem. While women already did the majority of the unpaid care work in households before the beginning of COVID-19 pandemic, recent studies show that this load has increased dramatically due to the crisis. The negative effects on women and families are likely to last for several more years. What we usually call the “economy” would not be able to function without the (often unrecognized) work ensured by the care economy: providing daily living, cooking, upbringing children, etc. (Power, 2020; Ramakrishnan, 2020a,b). The care economy globally, comprising both paid and unpaid care work, underpins and sustains the market economy. Unpaid work accounts for 16.4 billion hours a day, three-quarters performed by women—as the International Labour Organization reports, this is equivalent to two billion jobs. Paid care work, 11.5% of global employment, encompasses 381 million workers, two-thirds of whom are women (Sadasivam, 2020).

The research carried out by The European Foundation for the Improvement of Living and Working Conditions Agency before the COVID-19 emergency situation show an unequal distribution of paid and unpaid work between men and women in the EU countries. This is especially true for families with pre-school children. It was concluded that long working hours have a negative impact on work–life balance, and both men and women report that long working hours reduce their opportunities to combine work and family responsibilities. The particularly negative influence of work on work–life balance tend to be focused in the life stage of employees when they bring up pre-school children. This period usually coincides with a reduction in working hours for working mothers and an increase for working fathers, while both sexes would prefer to work shorter hours during this period of life (Eurofound, 2017).

Remote working during the COVID-19 pandemic was more complicated than remote working under normal circumstances, as it was compulsory rather than voluntary, often full-time, rather than part-time or casual. In addition, surveys conducted within some studies suggest that there is also a positive experience of working remotely from home (Cartmill, 2020; Gálvez et al., 2020; Uresha, 2020). An approach which facilitates work–life balance of employees and provides for organizing and evaluating remote working according to the results should be supported, rather than focusing on the number of hours or specific work schedules. Defining clear requirements for specific results to be achieved, employees are better prepared to manage their time and tasks, thus effectively balancing their work responsibilities with personal life, including family responsibilities. One of the most significant problems faced by employees working remotely during the pandemic is the conflict between work and private life, as they experience a blurred line between work and private life. Defining the boundaries between work and private life is always a topical issue in the case of remote working, but it is particularly problematic due to the unique circumstances of the pandemic (ILO, 2020).

The crisis during COVID-19 has shattered the notion that paid work and personal life are two completely different areas, and there appeared a myth that employees always can and must be available to the employer to perform their work-related functions (ILO, 2020).

Trends in the labor market already even before the COVID-19 pandemic suggested that employers face difficulties and significant challenges in attracting the workforce required by companies, both in general and at different levels of qualifications and positions. Often employers mentioned flexible working hours and various social guarantees in their job advertisements as benefits that could be of interest to potential employees. How the employees in Latvia evaluated these additional benefits was studied in a Eurobarometer survey on work–life balance. In Latvia, the survey was conducted by the research agency Kantar in the framework of the Flash Eurobarometer 47 from June 26 to 30, 2018, surveying 1,000 Latvians aged 15 and over. In total, the survey was conducted in 28 EU Member States (26,578 respondents). 65% of those Latvians for whom flexible working hours were not available wanted to use the possibility of flexible working hours or adjust the start and end time of work. About one in five respondents (18%) wanted to take the opportunity to work from home, 1 in 10 (12%)—wanted to take the opportunity to work part-time. The results reveal that the majority or 73% of Latvian employees (self-employed, hired workers, and manual workers) were generally satisfied with their work–life balance, but the average level of overall satisfaction among the EU employees was significantly higher at 79%. Viewing the work–life balance at the Baltic level, the satisfaction levels of Estonian and Lithuanian population were also significantly higher than in Latvia and above the European average level with 80% of Lithuanian and 81% of Estonian population satisfied with their work–life balance (Kantar, 2018).

The results of the study conducted by this agency in August 2020 show that during the COVID-19 restrictions in the spring, for Latvian inhabitants it was comparatively the most difficult to reconcile work and private life as well as taking care of their families. There was a tendency for women with low average family income (up to 300 euro a month) and those with children in the family (especially if more than four) to report relatively more often that it was difficult to balance remote working with taking care of children and parenting. It is likely that this was largely influenced by children’s distance learning process and changes in the daily routines, which were mainly taken care of by women in parallel with their job responsibilities regardless of work schedule (onsite or remotely) (Kantar, 2020).

In addition, there should be noted a number of negative features of remote working that can potentially affect work–life balance as well as the psycho-emotional state of employees and consequently work efficiency. The available data suggest that remote working makes it more difficult for workers to comply with EU directives (Directive (EU) 2003/88/EK of the European Parliament and of the Council, 2003; Directive (EU) 2019/1152 of the European Parliament and of the Council, 2019; Directive (EU) 2019/1158 of the European Parliament and of the Council, 2019) and the terms specified in the national regulatory enactments which are related to rest and the maximum weekly working time, especially in connection with unpaid overtime work. During remote working, the boundaries between work and private time become more blurred, making it difficult to distinguish between working time and rest periods (Eurofound, 2020b).

Eurofound research shows that while the use of remote working and flexible working allows workers to better balance working hours and leisure time, it can also have a negative impact on work–life balance, as teleworkers or flexible workers are more likely to work longer hours and overtime, they have fewer rest periods and less predictable and irregular schedules (except for night work). The reasons for this are heavy workload, accessibility outside normal working hours, frequent interruptions, and (to some extent) a degree of autonomy (Eurofound, 2019).

## Methodology

This article is based on the results of a large-scale research conducted within the framework of the Latvian National Research Programme Project—Life with COVID-19: Evaluation of Overcoming the Coronavirus Crisis in Latvia and Recommendations for Societal Resilience (CoLife), one of the goals of which was to evaluate the influence of the COVID-19 restriction period in the spring of 2020 on changes in forms of employment and employees’ ability to combine work responsibilities and private life from different aspects (employees’ gender, age, region of residence, presence of children under 18 in the household, etc.); identify factors promoting and hindering the balance of private life on the part of both employees and employers. The research also assessed the role of employers in promoting work–life balance for employees, including remote workers, taking into account that during the COVID-19 emergency situation, most employees needed to perform both work and family responsibilities at the same time. In particular, this article reflects only a small part of the results of the research carried out within the project.

In order to obtain the data required for the research, a structured survey of the employed Latvian population was conducted. The questionnaire was coordinated with the Labour Relations and Labour Protection Policy Department of the Ministry of Welfare of the Republic of Latvia. The survey was disseminated via an internet link on publicly available websites, social networks as well as through direct e-mails from September 28, 2020 until October 27, 2020. At the beginning of the survey, filtering questions were applied to recruit only paid workers who were employed during the previous year. The following exclusion criteria were used: working without salary in family businesses, working without salary on family farm, being on maternity leave, being unemployed persons, being only retired persons, being housewives, being only school-children, or students during the survey period.

The so-called snowball effect and social network advertising were used as a method of disseminating the survey, adapting the advertisement to maximize the recruitment of the missing groups of respondents. While designing the survey, the survey sample size was calculated, using 5% margin error, 99% confidence intervals, 50% response rate, and 892,100 employed persons in Latvia in the second quarter of 2020 (Central Statistical Bureau of Latvia, 2020), resulting in 663 persons. To increase the probability of finding statistically significant results and taking into account the planned time frame of the survey, the authors decided to make the web-link available one full calendar month or until the moment when there will be 1,000 fully filled answers, whichever will occur first. In this case, the link to the web-survey was locked on the next morning of workday after 1,000 respondents have answered all of the survey questions. In total, 1,823 people took part in the survey, but considering that the survey was relatively long only 1,006 respondents answered all the questions (response rate—55.2%). A detailed description of the sample of respondents who answered all of the questions is available in Table 1. However, this study analyzes the responses of all employed respondents who answered the certain survey question, which provides the highest coverage of the number of respondents.

**TABLE 1:** Distribution of the total study sample, taking into account those respondents who answered all the questionnaire questions, *n* (%).

Gender		
Male	204 (20.3%)	
Female	802 (79.7%)	
**Age groups (years)**		
18–24	35 (3.5%)	
25–34	203 (20.2%)	
35–44	298 (29.6%)	
45–54	270 (26.8%)	
55–63	169 (16.8%)	
64+	31 (3.1%)	
**The presence of children under 18 in the household**		
Yes	441 (44.0%)	196 (19.5%)* have children in the age group of 0–6 (preschool), in total 240 children
		490 (48.7%)* have children in the age group 7–18 (school), in total 533 children
No	562 (56.0%)	
**Worked remotely during the first wave COVID-19 emergency situation**		
Yes	486 (48.3%)	
No	520 (51.7%)	

*These may include respondents with children of both age groups in the household.

At the beginning of the web-survey, written information on the purpose of the study was provided, therefore, participants by voluntary proceeding to the questions agreed to participate in the survey. The answers provided by the survey respondents are confidential and were analyzed in an aggregated way.

The data of the web-survey were collected and managed using REDCap (Research Electronic Data Capture) tool for electronic data collection and compilation. REDCap is a secure, web-based software platform designed to support data collection for research. The study applied a non-probability sampling method which is one of the limitations of this study. To overcome it at least partly and to obtain data that is representative for the demographic profile of the working population in Latvia, data were weighted by age crossed with gender (in 12 age-gender combinations). Weighting targets included 2020 year third quarter population estimates from the Central Statistical Bureau of Latvia by age groups and gender. Data analysis was performed using quantitative methods. All the survey data were weighted and analyzed with the data processing programme IBM SPSS (version 26) and visualized using MS Excel.

This article includes analysis of the answers of Latvian employed respondents to certain survey questions about their work–life balance during the emergency situation caused by the first wave of the COVID-19 in the spring of 2020 by gender, age group of respondents as well as depending on the presence of minor children in the respondents’ households. The SPSS Compare Means—The Independent Samples T-Test Method was used to analyze respondents’ responses depending on their gender and presence of children under 18 in the household, which is used in cases where only two variables (groups) are compared. In turn, the SPSS Compare Means—One-Way ANOVA Method was used to analyze the respondents’ responses by age groups, which is used when more than two variables (groups) are compared. Respondents’ answers to the last question viewed in the article were analyzed using the SPSS Frequencies distributions method.

## Research Outcomes

Although this research was conducted in the second half of 2020, it focused on earlier developments—the emergency situation caused by the COVID-19 pandemic in Latvia in the first half of 2020, or the period when the Latvian government decided to reduce the spread of COVID-19 on March 12, 2020, making a decision to declare a state of emergency in the country, which lasted until June 10, the so-called first wave of COVID-19. During this time, education process in schools took place remotely, as did the work of state and local government institutions where it was possible. The availability of kindergarten services was significantly limited. Likewise the private sector had to organize work remotely as much as possible.

Only those respondents who were employed during the first wave of COVID-19 could participate in the survey. The total number of respondents who answered all the questions of the web-survey is 1,006 employees. Of these, 79.7% are women and 20.3% are men; 44.0% of them have minor children in the household, 48.3% of the respondents worked remotely during the COVID-19 emergency situation.

The results of the study are summarized in Tables 2–6, which show the analysis of answers provided by unweighted sample of respondents and weighted sample of respondents. Further in the text of the article, only the analysis of weighted sample of respondents’ answers was considered.

**TABLE 2:** Analysis of respondents’ answers to the question “When working remotely, did you feel that family life affects your ability to perform work responsibilities?” (*n* = 522).

Question^a^				Multiple choice answers^b^		Values	
*When working remotely, did you feel that family life* *influences your ability to perform work responsibilities?*				Yes		1	
				No		2	

		**Unweighted sample of respondents**			**Weighted sample of respondents**		
**Variables**		**Mean**	**Significance, *p***	**Statistical significance**	**Mean**	**Significance, *p***	**Statistical significance**

Gender	Male	1.45	Sig. (2-tailed) = 0.580	Sig. (2-tailed) > 0.05 Group results are not statistically significantly different	1.43	Sig. (2-tailed) = 0.954	Sig. (2-tailed) > 0.05 Group results are not statistically significantly different
	Female	1.42			1.43		
The presence of children under 18 in the household	Yes	1.22	Sig. (2-tailed) < 0.001	Sig. (2-tailed) ≤ 0.05 Group results are statistically significantly different	1.21	Sig. (2-tailed) < 0.001	Sig. (2-tailed) ≤ 0.05 Group results are statistically significantly different
	No	1.62			1.61		
Age groups	18–24	1.54	Sig. < 0.001	Sig. ≤ 0.05 Group results are statistically significantly different	1.66	Sig. < 0.001^c^	Sig. ≤ 0.05 Group results are statistically significantly different
	25–34	1.42			1.38		
	35–44	1.29			1.29		
	45–54	1.52			1.55		
	55–63	1.57			1.54		
	64+	1.62			1.53		

*^*a*^Only respondents who worked remotely during the COVID-19 emergency situation could answer this question.*

*^*b*^The answer option “Hard to say” was not analyzed.*

*^*c*^Statistical significance *p* is observed between the following age groups: *p* = 0.038—18–24 years and 25–34 years; *p* = 0.005—18–24 years and 35–44 years; *p* = 0.019—25–34 years and 45–54 years; *p* = 0.039—25–34 years and 55–63 years; *p* < 0.001—35–44 years and 45–54 years; *p* = 0.001—35–44 years and 55–63 years.*

The survey included a number of questions about the working and home conditions of employees, which directly and indirectly affect work–life balance.

Analysis of respondents’ answers to the question *“When working remotely, did you feel that family life affects your ability to perform work responsibilities?”* is available in Table 2. Only respondents who worked remotely during the first COVID-19 emergency situation could answer this question (*n* = 522).

The data in Table 2 show that the difference between the mean values of the answers of the respondents by gender is -0.003, and the results of the group answers do not differ statistically significantly, as evidenced by the statistical significance index *p* = 0.954 (>0.05). It can be concluded that the answers provided by the respondents to this question do not differ significantly by gender.

Analyzing the answers provided by the respondents depending on whether or not there are children under 18 in their households, it is evident that the difference between the mean values of the answers provided by the respondents is -0.398, and the results of the group answers are statistically significantly different, as evidenced by the statistical significance index *p* < 0.001 (≤0.05). The mean answer value 1.21 for respondents with children under the age of 18 in the household means that when working remotely, they generally felt that family life affected their ability to perform their work responsibilities during the COVID-19 emergency situation, while the mean answer value 1.61 for respondents, who have no children under the age of 18 in the household, indicates that they largely did not feel the impact of family life on their ability to perform work responsibilities. It can be concluded that the presence of children under the age of 18 in the household is an important factor that negatively affects the work–life balance.

Analyzing the answers of the respondents by age groups, it can be noted that the results of the group answers are statistically significantly different between the following age groups: the statistical significance index *p* = 0.038 (≤0.05) exists between the respondent age groups of 18–24 and 25–34 years; the statistical significance index *p* = 0.005 (≤0.05) exists between the respondent age groups of 18–24 and 35–44 years; *p* = 0.019 (≤0.05)—between the respondent age groups 25–34 years and 45–54 years; *p* = 0.039 (≤0.05)—between the respondent age groups 25–34 years and 55–63 years; *p* < 0.001 (≤0.05)—between the respondent age groups 35–44 years and 45–54 years; and *p* = 0.001 (≤0.05)—between the respondent age groups 35–44 years and 55–63 years. There are no statistically significant differences in the results of the answers of the respondents of other age groups. The mean values of the answers of respondents in the age groups of 25–34 years and 35–44 years (1.38 and 1.29, respectively) show that mostly teleworkers of this age felt that family life affects their ability to perform work responsibilities, while the mean values of the answers of the respondents of other age groups show the opposite. It can be concluded that the respondents aged 25–44 felt work-life imbalance the most during the COVID-19 emergency. One of the reasons could be that this is the age when respondents have pre-school and school-age children who needed parental supervision and additional support during the COVID-19 emergency, especially for distance learning. It should be noted that 79.3% of respondents in the age group 35–44 years have at least 1 child under the age of 18 in the household, which is the highest indicator among all age groups.

Summarizing the teleworkers’ answers to the question of whether they felt that family life affected their ability to perform work responsibilities while working remotely, it can be concluded that the respondents of both genders aged 25–44 with children in the household under the age of 18 felt it.

The analysis of the respondents’ answers to the question *“Taking into account your remote working experience during the COVID-19 emergency situation, please evaluate how the balance between your work and private life has changed”* is available in Table 3. Only respondents who worked remotely during the COVID-19 emergency situation could answer this question (*n* = 512).

**TABLE 3:** Analysis of respondents’ answers to the question “Taking into account your remote working experience during the COVID-19 emergency situation, please evaluate how the balance between your work and private life has changed” (*n* = 512).

Question^a^				Multiple choice answers^b^		Values	
*Please evaluate how your work–life balance has changed*, *considering your remote working experience during the COVID-19 emergency situation*				Improved		1	
				Did not change		2	
				Became worse		3	

		**Unweighted sample of respondents**			**Weighted sample of respondents**		
**Variables**		**Mean**	**Significance, *p***	**Statistical significance**	**Mean**	**Significance, *p***	**Statistical significance**

Gender	Male	1.93	Sig. (2-tailed) = 0.191	Sig. (2-tailed) > 0.05 Group results are not statistically significantly different	1.95	Sig. (2-tailed) = 0.157	Sig. (2-tailed) > 0.05 Group results are not statistically significantly different
	Female	2.07			2.06		
The presence of children under 18 in the household	Yes	2.13	Sig. (2-tailed) = 0.031	Sig. ≤ 0.05 Group results are statistically significantly different	2.07	Sig. (2-tailed) = 0.239	Sig. (2-tailed) > 0.05 Group results are not statistically significantly different
	No	1.96			1.97		
Age groups	18–24	2.23	Sig. = 0.313	Sig. > 0.05 Group results are not statistically significantly different	2.17	Sig. = 0.493	Sig. > 0.05 Group results are not statistically significantly different
	25–34	1.97			2.01		
	35–44	2.14			2.06		
	45–54	1.94			1.91		
	55–63	2.00			1.98		
	64+	2.15			2.30		

*^*a*^Only respondents who worked remotely during the COVID-19 emergency situation could answer this question.*

*^*b*^The answer option “Hard to say” was not analyzed.*

The data in Table 3 show that the difference between the mean values of the answers provided by the respondents by gender is -0.112, and the results of the group answers have no statistically significant differences, as evidenced by the statistical significance index *p* = 0.157 (>0.05). It can be concluded that the answers provided by the respondents to this question do not differ significantly by gender.

Analyzing the answers provided by the respondents depending on whether or not there are children under the age of 18 in their households, it is evident that the difference between the mean values of the answers provided by the respondents is 0.095, and the results of the group answers have no statistically significant differences, as evidenced by the statistical significance index *p* = 0.239 (>0.05). It means that the answers of the respondents to this question do not differ significantly depending on the presence of children under the age of 18 in their households.

Despite the fact that the mean values of the respondents’ answers differ across age groups, the statistical significance index *p* = 0.493 (>0.05) suggests that these results are not statistically significantly different. It can be concluded that the answers of the respondents to this question do not differ significantly across age groups.

Summarizing the data analysis in the Table 3, it can be concluded that the respondents’ answers to the question of how their work–life balance changed when working remotely during the COVID-19 emergency situation did not differ significantly by gender and age group, as well as did not vary depending on whether or not there are children under the age of 18 in the respondents’ households.

Analysis of respondents’ answers to the question *“Is it important for you be able to disconnect from digital devices outside working hours/after completing the assigned work tasks?”* is available in the Table 4. Only the respondents who worked remotely during the COVID-19 emergency situation could answer this question (*n* = 515).

**TABLE 4:** Analysis of respondents’ answers to the question “Is it important for you be able to disconnect from digital devices outside working hours/after completing the assigned work tasks?” (*n* = 515).

Question^a^				Multiple choice answers^b^		Values	
*Is it important for you to be able to disconnect from* *digital devices outside working hours/after completing the* *assigned work tasks?*				Yes		1	
				No		2	

		**Unweighted sample of respondents**			**Weighted sample of respondents**		
**Variables**		**Mean**	**Significance, *p***	**Statistical significance**	**Mean**	**Significance, *p***	**Statistical significance**

Gender	Male	1.34	Sig. (2-tailed) = 0.065	Sig. (2-tailed) > 0.05 Group results are not statistically significantly different	1.31	Sig. (2-tailed) = 0.106	Sig. (2-tailed) > 0.05 Group results are not statistically significantly different
	Female	1.23			1.23		
The presence of children under 18 in the household	Yes	1.24	Sig. (2-tailed) = 0.833	Sig. (2-tailed) > 0.05 Group results are not statistically significantly different	1.27	Sig. (2-tailed) = 0.899	Sig. (2-tailed) > 0.05 Group results are not statistically significantly different
	No	1.25					
					1.26		
Age groups	18–24	1.08	Sig. = 0.092	Sig. > 0.05 Group results are not statistically significantly different	1.06	Sig. = 0.169^c^	Sig. > 0.05 Group results are not statistically significantly different
	25–34	1.17			1.23		
	35–44	1.22			1.24		
	45–54	1.33			1.32		
	55–63	1.29			1.31		
	64+	1.31			1.43		

*^*a*^Only the respondents who worked remotely during the COVID-19 emergency situation could answer this question.*

*^*b*^The answer option “Hard to say” was not analyzed.*

*^*c*^Statistical significance *p* is observed between the following age groups: *p* = 0.044—18–24 years and 45–54 years; *p* = 0.050—18–24 years and 55–63 years; *p* = 0.028—18–24 years and 64+ years.*

The data in Table 4 show that the results of the group answers do not differ statistically significantly by gender, as evidenced by the statistical significance index *p* = 0.106 (>0.05). It can be concluded that the answers provided by the respondents to this question do not differ significantly by gender.

Analyzing the answers provided by the respondents depending on whether or not there are children under 18 in their households, it is obvious that the results of the answers of both groups are not statistically significantly different, as evidenced by the statistical significance index *p* = 0.899 (>0.05). It means that the answers of the respondents to this question do not differ significantly depending on the presence of children under the age of 18 in their households.

As regards the fact that in general the results of the respondents’ answers across age groups are not statistically significantly different, which is evidenced by the statistical significance index *p* = 0.169 (>0.05), it should be noted that statistical significance *p* = 0.044 (≤0.05) is observed between the age groups of 18–24 and 45–54 years; *p* = 0.05 (≤0.05)—between the age groups 18–24 and 55–63 years; and *p* = 0.028—between the age groups 18–24 and 64+ years. Considering the mean answer values for respondents in the age groups of 18–24, 25–34, and 35–44 years (1.06, 1.23, and 1.24, respectively), it can be concluded that for these teleworkers the ability to disconnect from digital devices outside working hours/after completion of the assigned work tasks during the COVID-19 emergency situation was more important when for respondents in the age groups of 45+.

Summarizing the answers of teleworkers to the question of whether it was important for them to be able to disconnect from digital devices outside working hours/after completing the assigned work tasks, it can be concluded that the opportunity to disconnect was most important for respondents aged 18–44, regardless of their gender and the presence of children under 18 in the household.

Analysis of respondents’ answers to the question *“Did you incur any additional housework during the COVID-19 emergency situation?”* is available in Table 5. All the respondents could answer this question, irrespective of whether or not they worked remotely during the COVID-19 emergency situation (*n* = 1,049).

**TABLE 5:** Analysis of respondents’ answers to the question “Did you incur any additional housework during the COVID-19 emergency situation?” (*n* = 1,049).

Question^a^				Multiple choice answers^b^		Values	
*Did you incur additional housework* *during the COVID-19 emergency?*				Yes		1	
				No		2	

		**Unweighted sample of respondents**			**Weighted sample of respondents**		
**Variables**		**Mean**	**Significance, *p***	**Statistical significance**	**Mean**	**Significance, *p***	**Statistical significance**

Gender	Male	1.78	Sig. (2-tailed) < 0.001	Sig. (2-tailed) ≤ 0.05 Group results are statistically significantly different	1.77	Sig. (2-tailed) = 0.002	Sig. (2-tailed) ≤ 0.05 Group results are statistically significantly different
	Female	1.66			1.68		
The presence of children under 18 in the household	Yes	1.50	Sig. (2-tailed) < 0.001	Sig. (2-tailed) ≤ 0.05 Group results are statistically significantly different	1.60	Sig. (2-tailed) < 0.001	Sig. (2-tailed) ≤ 0.05 Group results are statistically significantly different
	No	1.82			1.81		
Age groups	18–24	1.71	Sig. < 0.001	Sig. ≤ 0.05 Group results are statistically significantly different	1.61	Sig. = 0.006^c^	Sig. ≤ 0.05 Group results are statistically significantly different
	25–34	1.73			1.74		
	35–44	1.54			1.66		
	45–54	1.71			1.73		
	55–63	1.81			1.81		
	64+	1.74			1.69		

*^*a*^All the respondents could answer his question, irrespective of whether or not they worked remotely during the COVID-19 emergency situation.*

*^*b*^The answer option “Hard to say” was not analyzed.*

*^*c*^Statistical significance *p* is observed between the following age groups: *p* = 0.004—18–24 years and 55–63 years; *p* = 0.045—25–34 years and 35–44 years; *p* < 0.001—35–44 years and 55–63 years.*

The data in Table 5 suggest that the results of the answers of the respondent groups are statistically significantly different, as evident from the statistical significance index *p* = 0.002 (≤0.05), which means that the answers of the respondents to this question differ by gender. As regards the mean value of the respondents’ answers, it is obvious that the difference between the mean values of the respondents’ answers by gender is small 0.093; however, the mean value of women’s answers of 1.68 indicates that women slightly more often than men indicated that they incurred additional responsibilities in the household during the COVID-19 emergency.

Analyzing the answers provided by the respondents depending on whether or not there are children under 18 in their households, it is evident that the difference between the mean values of the answers provided by the respondents is -0.202, and the results of the group answers are statistically significantly different, as evidenced by the statistical significance index *p* < 0.001 (≤0.05). The mean answer value of 1.60 for respondents who have children under the age of 18 in their households means that there were more respondents in this group who believed that they incurred additional housework during the COVID-19 emergency situation, while for respondents who have no children under the age of 18 in their households, the mean answer value is 1.81, indicating that there were more respondents in this group who believed that they did not incur additional housework. It enables a conclusion that the additional burden of responsibilities is related to the presence of children under the age of 18 in the household, which can have a negative impact on work–life balance.

Analyzing the answers of the respondents by age groups, it can be noted that the results of the group answers are statistically significantly different between the following age groups: the statistical significance index *p* = 0.004 (≤0.05) exists between the age groups of respondents who are 18–24 and 55–63 years old; statistical significance index *p* = 0.045 (≤0.05)—between the age groups of respondents who are 25–34 and 35–44 years old; and *p* < 0.001 (≤0.05)—between the age groups of respondents who are 35–44 and 55–63 years old. There are no statistically significant differences in the results of the answers of the respondents of other age groups. It should be noted that the mean values of the answers of the respondents in the age group of 18–24 and 35–44 years are the lowest—1.61 and 1.66, respectively, which means that these respondents noted more that they incurred additional responsibilities in the household during the COVID-19 emergency situation.

Summarizing the respondents’ answers about additional housework incurred during the COVID-19 emergency, it can be concluded that those employees who indicated the emergence of additional responsibilities in the household are of both genders (but slightly more often women) aged 18–24, who have children under the age of 18 in the household.

The analysis of the respondents’ answers to the question *“Please evaluate how your workload changed in terms of the household responsibilities during the COVID-19 emergency situation”* is available in Table 6. All the respondents could answer his question, irrespective of whether or not they worked remotely during the COVID-19 emergency situation (*n* = 1,103).

**TABLE 6:** Analysis of respondents’ answers to the question “Please evaluate how your workload changed in terms of the household responsibilities during the COVID-19 emergency situation” (*n* = 1,103).

Question^a^				Multiple choice answers^b^		Values	
*Please evaluate how your workload changed in* *terms of the household responsibilities during the COVID-19 emergency situation*				Significantly reduced		1	
				Slightly reduced		2	
				Did not change		3	
				Slightly increased		4	
				Significantly increased		5	

		**Unweighted sample of respondents**			**Weighted sample of respondents**		
**Variables**		**Mean**	**Significance, *p***	**Statistical significance**	**Mean**	**Significance, *p***	**Statistical significance**

Gender	Male	3.28	Sig. (2-tailed) < 0.001	Sig. (2-tailed) ≤ 0.05 Group results are statistically significantly different	3.30	Sig. (2-tailed) < 0.001	Sig. (2-tailed) ≤ 0.05 Group results are statistically significantly different
	Female	3.65			3.60		
The presence of children under 18 in the household	Yes	3.94	Sig. (2-tailed) < 0.001	Sig. (2-tailed) ≤ 0.05 Group results are statistically significantly different	3.69	Sig. (2-tailed) < 0.001	Sig. (2-tailed) ≤ 0.05 Group results are statistically significantly different
	No	3.30			3.29		
Age groups	18-24	3.61	Sig. < 0.001	Sig. ≤ 0.05 Group results are statistically significantly different	3.64	Sig. < 0.001^c^	Sig. ≤ 0.05 Group results are statistically significantly different
	25-34	3.56			3.46		
	35-44	3.86			3.63		
	45-54	3.48			3.39		
	55-63	3.32			3.30		
	64 +	3.29			3.30		

*^*a*^All the respondents could answer his question, irrespective of whether or not they worked remotely during the COVID-19 emergency situation.*

*^*b*^The answer option “Hard to say” was not analyzed.*

*^*c*^Statistical significance *p* is observed between the following age groups: *p* = 0.047—18–24 years and 45–54 years; *p* = 0.007—18–24 years and 55–63 years; *p* = 0.036—18–24 years and 64+ years; *p* = 0.026—25–34 years and 35–44 years; *p* = 0.048—25–34 years and 55–63 years; *p* = 0.002—35–44 years and 45–54 years; *p* < 0.001—35–44 years and 55–63 years; *p* = 0.011—35–44 years and 64+ years.*

The data in Table 6 show that the results of the answers of the respondent groups are statistically significantly different by gender, which is evidenced by the statistical significance index *p* < 0.001 (≤0.05), which means that the answers of the respondents to this question differ by gender. As regards the mean value of the respondents’ answers, it is evident that the difference between the mean values of the answers given by the respondents by gender is -0.308, but the mean value of women’s answers is 3.60, indicating that women were more likely than men to note increase in the load related to household responsibilities during the COVID-19 emergency situation, while the mean answer value of 3.30 for men means that men were more likely to note that their load of housework during the COVID-19 emergency situation did not change.

Analyzing the answers provided by the respondents depending on whether or not there are children under the age of 18 in their households, it is evident that the difference between the mean values of the answers provided by the respondents is 0.399, and the results of the group answers are statistically significantly different, as evidenced by the statistical significance index *p* < 0.001 (≤0.05). The mean answer value of 3.69 for respondents with children under the age of 18 in the household clearly indicated that this group of respondents generally noted a slight increase in the load of housework during the COVID-19 emergency, while for the respondents who have no children under the age of 18 in the household, the mean value of the answers is 3.29, indicating that there were more respondents in this group who believed that their workload did not change. It enables a conclusion that the increase in workload concerning household responsibilities is closely related to the presence of children under the age of 18 in the household, which may have a negative impact on work–life balance.

Analyzing the answers of the respondents by age groups, it can be noted that the results of the group answers are statistically significantly different between the following age groups: the statistical significance index *p* = 0.047(≤0.05) exists between the age groups of respondents who are 18–24 and 45–54 years old; statistical significance index *p* = 0.007 (≤0.05)—between the age groups of respondents who are 18–24 and 55–63 years old; *p* = 0.036 (≤0.05)—between the age groups 18–24 years and 64+ years; *p* = 0.026 (≤0.05)—between the age groups 25–34 years and 35–44 years; *p* = 0.048 (≤0.05)—between the age groups 25–34 years and 55–63 years; *p* = 0.002 (≤0.05)—between the age groups 35–44 years and 45–54 years; *p* < 0.001 (≤0.05)—between the age groups 35–44 years and 55–63 years; and *p* = 0.011 (≤0.05)—between the age groups 35–44 years and 64+ years. There are no statistically significant differences in the results of the answers of the respondents of other age groups. It should be noted that the mean values of the answers of the respondents in the age group of 18–24 and 35–44 years are the highest—3.64 and 3.63, respectively, which means that these respondents noted increase in the load related to household responsibilities during the COVID-19 emergency situation.

Summarizing the answers of the respondents about the changes in the workload of their housework during the COVID-19 emergency situation, it can be concluded that more often women, respondents aged 18–24 and 35–44 and those who have children under the age of 18 in their households indicated to increase in workload.

All the respondents, irrespective of whether or not they worked remotely during the COVID-19 emergency situation, could answer the question *“Please mark what changes were made to your household’s daily routine during COVID-19 to enhance work–life balance?”*, choosing one of the following answers:

1. There were changes in work pattern (for example, it was coordinated with other family members, a flexible, result-oriented work pattern was applied, etc.);
2. Redistribution of household responsibilities among household members (for example, house cleaning, doing homework with children, babysitting, etc.);
3. Involvement of assistants in household maintenance and tidying work (for example, relatives or friends);
4. Use of outsourced services (for example, delivery of food and ready meals, babysitter services, etc.);
5. The place of residence was changed (for example, moving from the city to the countryside);
6. Other changes; and
7. Hard to say.

**FIGURE 1:**
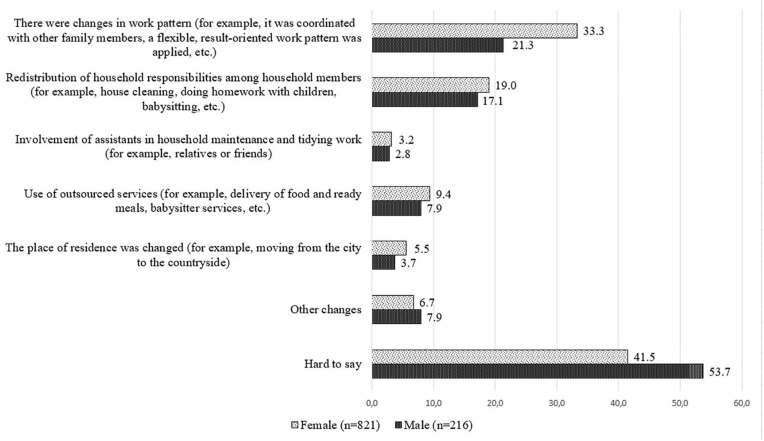
Distribution of answers of the employed respondents to the question “Please mark what changes were made to your household’s daily routine during COVID-19 to enhance work–life balance?”; gender; %.

Analyzing the data by gender, they suggest that in general women were more likely than men to choose an answer from 1 to 5. On the other hand, men pointed to “*other changes*” in the daily routine to enhance work–life balance, mostly stating that nothing had changed for them in the daily routine concerning housework. Only one man indicated emigration as other change in the daily routine. It should be noted that the majority of men found it difficult to answer this question—53.7%, compared to women—41.5%. As *“other changes”* in the daily routine, women mostly mentioned that they were forced to help children in the distance learning process, had to look after their grandchildren, had to provide the elderly parents with food and other goods, had to spent more time on cooking at home (see Figure 1).

**FIGURE 2:**
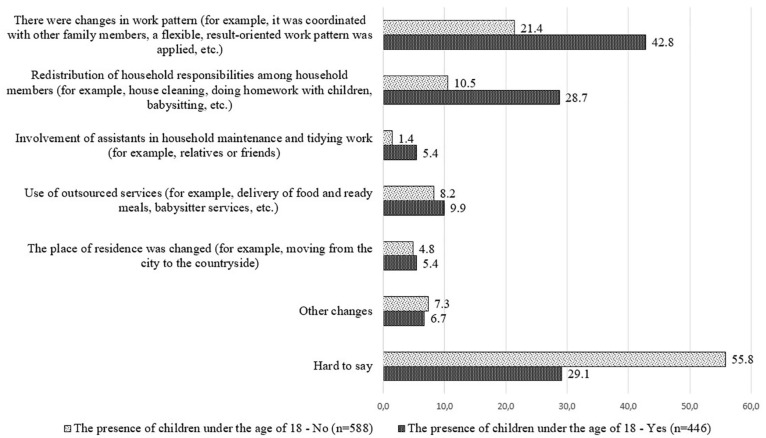
Distribution of answers of employed respondents to the question “Please mark what changes were made to your household’s daily routine during COVID-19 to enhance work–life balance?”; presence of children under the age of 18 in the household; %.

Analysis of the responses of the respondents depending on whether or not there are children under the age of 18 in their households, shows that the respondents with children under the age of 18 in their households were more likely to choose answers between 1 and 5. This means that in order to ensure balance between work and private life, these respondents needed to adjust the daily routine of their household members during the COVID-19 emergency situation to be able to perform both work duties and other household responsibilities, including childcare. In turn, those respondents who had no children under the age of 18 in the household more often referred to “*other changes*” that were made in the daily routine or chose the answer option “*hard to say*” (see Figure 2).

**FIGURE 3:**
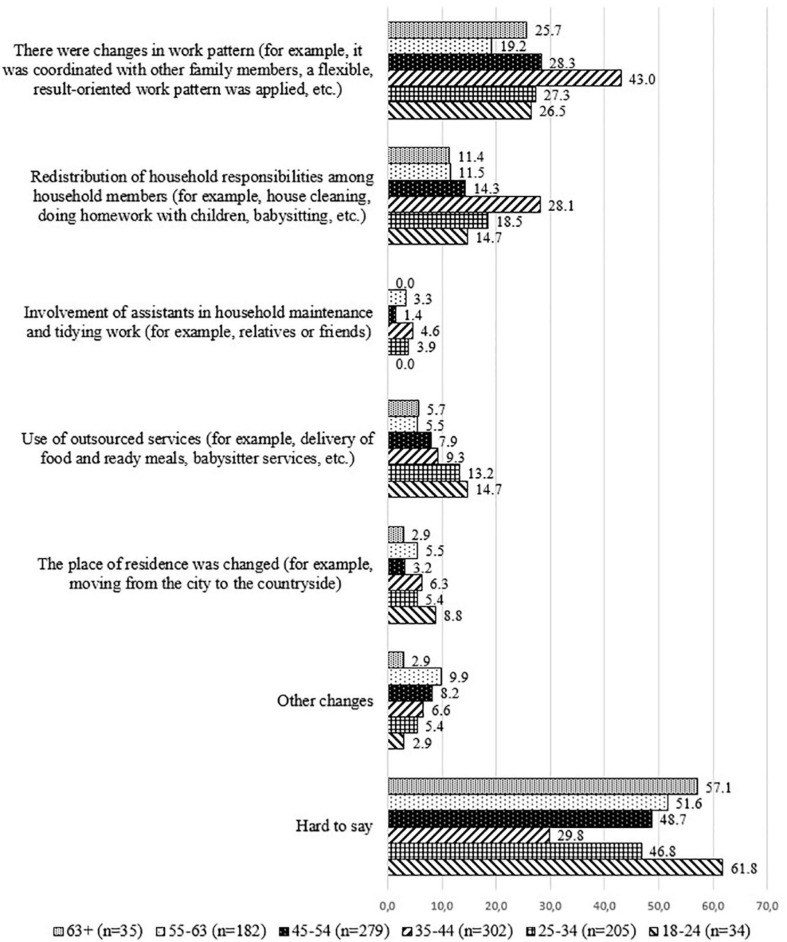
Distribution of answers of employed respondents to the question “Please mark what changes were made to your household’s daily routine during COVID-19 to enhance work–life balance?”; age groups; %.

Analyzing the obtained data depending on the age groups of the respondents, it can be concluded that the answer options 1 and 2 were mostly chosen by the employees aged from 35 to 44. This group of respondents chose the answer “*hard to say*” the least—29.8%. One of the reasons for this could be that this is the age when respondents have school-age children who needed parental supervision and additional support during the COVID-19 emergency situation, especially in the process of distance learning. It should be noted that no significant differences for the answer options from three to six by the respondent age groups were observed (see Figure 3).

Summarizing the information about the major changes in respondents’ households aimed at enhancing work–life balance during the COVID-19 emergency situation, it can be concluded that women aged 35–44 with children under the age of 18 in their households the most frequently involved in changes related to working patterns and redistribution of household responsibilities.

## Concluding Discussion

Evaluating the results of the survey, it can be concluded that the emergency situation caused by COVID-19 did not promote work–life balance of the employed in Latvia, especially of teleworkers, particularly if there were children under the age of 18 in the households.

So far, global research on employees’ work–life balance during the COVID-19 emergency situation has shown a similar trend, i.e., due to the COVID-19 pandemic the burden of household responsibilities on women significantly increased, especially if they had minor children. Del Boca et al. (2020) reveals that in Italy, working women with young children (particularly those aged 0–5) are most vulnerable and most aware of the difficult work–family balance. The COVID-19 crisis further increased the workload of women, resulting from both their housework and the occupation (Del Boca et al., 2020). In April 2020, a study conducted by Boston Consulting Group on the impact of the COVID-19 pandemic on working parents in the United States, United Kingdom, Italy, Germany, and France concluded that during the COVID-19 pandemic parents have almost doubled the time spent on education and household tasks, moreover, women per 31 percentage points more were shouldering a bigger share of the additional time spent on childcare and household tasks. Consequently, parents feel their ability of performance at work has dropped significantly, especially those with younger children (Boston Consulting Group, 2020). Chauhan (2020) in his research concluded that in India COVID-19 pandemic even more influenced the existing gender inequalities and increased both employed and unemployed women burden of unpaid work. In Spain, during the COVID-19 period in spring 2020 the amount of childcare and housework taken on by both parents increased considerably; however, women continued to shoulder most of the burden: a gender gap in parents’ shares of childcare and housework during the lockdown was found about 17 percentage points on average (Farré et al., 2020). It is obvious that the COVID-19 pandemic has affected employees of both genders, but it has affected women the most, in particular, if there are minor children in the household.

Summarizing the results of the survey of Latvian employees on the work–life balance of respondents and the problems related to ensuring it during the first wave of the COVID-19 emergency situation in the spring of 2020, a similar trend can be observed—employees of both genders felt that family life affected their ability to perform work duties remotely, especially the respondents aged 25–44, and those with children under the age of 18 in their household.

The deterioration of work–life balance during the COVID-19 emergency situation did not differ significantly by gender and age group, as well as did not vary depending on whether or not there are children under the age of 18 in the respondents’ households. However, an analysis of the responses of the unweighted sample of respondents suggests, that the presence of children under the age of 18 in the household affected the deterioration of work–life balance during the COVID-19 emergency situation.

For younger teleworkers aged 18–44, it was important to disconnect from digital devices outside working hours/after completing the assigned work tasks, regardless of their gender and the presence of children under 18 in the household. This is one of the crucial factors in ensuring work–life balance, especially for teleworkers.

The emergence of additional responsibilities in the household during the COVID-19 emergency situation was slightly more frequently indicated by women, as well as by employed respondents at the age of 18–24 and 35–44 who have children younger than 18 in the household. In general, employed women were more likely to point at changes in the household’s daily routine, such as modifying work patterns, redistributing household responsibilities among the household members, involving assistants in housework or using outsourced services to balance their work–private life during the COVID-19 emergency situation.

It can be concluded that the research hypothesis put forward that during the COVID-19 emergency situation all the groups of employees experienced difficulties in balancing work and private life is partially confirmed, i.e., employed women in the age group of 18–44 and the respondents with minor children in the household more often experienced difficulties in balancing work and private life.

The research limitations of our study are related to formation of selection amount, as only the people who had access to the internet could participate in the web-survey. As a result some groups of workers may be excluded from the sample by default (e.g., elderly, people living in remote areas, and people with low education and digital literacy). The same, so-called snowball, recruiting principle was used during the Eurofound survey “Living, working and COVID-19” (Eurofound, 2020a). In addition, the questionnaire was available only in Latvian, and it might have caused less response rate from the side of the Russian-speaking population. A non-probability sampling method which was used to gather survey data is another limitation of the study. The advantage of this method is the possibility to quickly gather information from respondents which was important because of the implementation requirements of the project “Life with COVID-19: Evaluation of Overcoming the Coronavirus Crisis in Latvia and Recommendations for Societal Resilience in the Future” (CoLife). To overcome this limitation at least partly and to obtain data that is representative of the demographic profile of the working population in Latvia, data was weighted by age crossed with gender (in 12 age-gender combinations). Weighting targets included 2020 year third quarter population estimates from Central Statistical Bureau of Latvia by age groups and gender. It should be noted that the seasonality impact was not studied in this article, which can also be considered as a limitation of the study.

This research examines a small part of the questions included in the survey, which was conducted within the framework of the Latvian National Research Programme Project “CoLife,” one of the goals of which was to assess the flexibility of work–life balance in different socio-demographic groups during the COVID-19 emergency situation in Latvia in the spring of 2020. The evaluation revealed that the risk group of employees most affected by the emergency situation of the first wave of COVID-19 in Latvia are middle-aged (35–44 years) women with children under the age of 18 in the household. Although only a small part of the range of issues covered by this research was addressed in this article, the result suggests a similar trend.

Although remote working is mentioned in scientific and practical research as one of the tools for work–life balance, it should be borne in mind that the daily private life, responsibilities and timing of employees with minor children had changed during the COVID-19 emergency situation. Considering that the emergency situation has encouraged the use of remote working, with the tendency to grow, it is essential to facilitate/ensure work–life balance for all employees, regardless of the type of employment and the form of working hours. As noted by Del Boca et al. working from home may have important consequences on gender gaps. On the one hand, a proper flexibility is desirable for better work–life balance of both men and women, it may result in better sharing of family work within the couple. On the other hand, if this becomes a female-dominated option, with men mostly working at the workplace and women working from home, a critical increase of unbalanced family work with majority of the work borne by women is observed (Del Boca et al., 2020).

It should be noted that work–life imbalance is one of the factors that negatively affects sustainability of work (the ability to work for up to the age of 60 or more), i.e., the physical and mental health and well-being of employees. Work–life balance contributes to increasing work efficiency and productivity, improving the health of employees, strengthening the highest motivation and loyalty of employees, etc. In order to promote a work–life balance for employees, employers must use such patterns of working hours that prevent negative influence on health and well-being of the employees, inclusion of family-friendly initiatives in personnel policies of the companies through collective agreements at the level of a sector or company. In turn, the government also needs to think about high-quality, accessible, family-friendly care infrastructure (i.e., childcare, care for the elderly people, care for people with special needs, and other public services).

Work–life balance requirements are highly dependent on the individual’s personal circumstances, such as the partner’s working hours and the presence of children or elderly dependents in the household. And these conditions change over a lifetime. This is particularly relevant in view of the current postponement of the retirement age and the increase in life expectancy in the world, which means that it will be significant to ensure a balance between work and long-term care for family members in the future. It is therefore important to promote the development of accessible, high-quality care services for children, the sick, people with disabilities, the elderly and other dependents, especially in the place of their residence, taking into account priorities and principles of social service policy (deinstitutionalisation and provision of a service primarily at or close to the person’s place of residence) so that as many employed people as possible can reconcile work and family life.

It should be added that the research of the impact of the COVID-19 pandemic on the work–life balance of employees in Latvia continues after the completion of the “CoLife” project. At the beginning of 2021, the authors organized a repeated web-survey of Latvian employees intending to study the changes in the situation and the adaptation of employees to *new-normal* conditions.

## Data Availability Statement

The datasets presented in this study can be found in online repositories. The names of the repository/repositories and accession number(s) can be found below: Zenodo https://doi.org/10.5281/zenodo.5090519.

## Ethics Statement

The studies involving human participants were reviewed and approved by the Research Ethics Committee of Riga Stradins University (July 23, 2020, No. 6-1/08/16). Written informed consent for participation was not required for this study in accordance with the national legislation and the institutional requirements.

## Author Contributions

JL, IM, and LM: methodology. JL, LL, and LP: formal analysis and investigation. JL: writing—original draft preparation. IM, IA, and IV: writing—review and editing. LM: supervision. All authors: conceptualization. All authors contributed to the study conception and design.

## Conflict of Interest

The authors declare that the research was conducted in the absence of any commercial or financial relationships that could be construed as a potential conflict of interest.

## Publisher’s Note

All claims expressed in this article are solely those of the authors and do not necessarily represent those of their affiliated organizations, or those of the publisher, the editors and the reviewers. Any product that may be evaluated in this article, or claim that may be made by its manufacturer, is not guaranteed or endorsed by the publisher.
